# Prediction of pre-eclampsia by using radiomics nomogram from gestational hypertension patients

**DOI:** 10.3389/fnins.2022.961348

**Published:** 2022-08-05

**Authors:** Xue-Fei Liu, Jing-Jing Lu, Meng-Die Li, Ying Li, An-Rong Zeng, Jin-Wei Qiang

**Affiliations:** Department of Radiology, Jinshan Hospital of Fudan University, Shanghai, China

**Keywords:** pre-eclampsia, gestational hypertension, magnetic resonance imaging, radiomics, nomogram

## Abstract

**Background:**

Pre-eclampsia (PE) is the main cause of death in maternal and prenatal morbidity. No effective clinical tools could be used for the prediction of PE. A radiomics nomogram based on diffusion-weighted imaging (DWI) and apparent diffusion coefficient (ADC) maps was established to predict PE from gestational hypertension (GH).

**Materials and methods:**

A total of 138 patients with hypertensive disorders of pregnancy were continuously enrolled in the study prospectively, namely, 58 patients with PE and 80 patients with GH. The patients were randomly divided into a training cohort (*n* = 97) and a test cohort (*n* = 41). Radiomics features were extracted from DWI and ADC maps. The radiomics signature was constructed using a least absolute shrinkage and selection operator (LASSO) algorithm in the training cohort. A radiomics nomogram was developed by combining the radiomics signature with the selected clinical risk factors. The area under the receiver operating characteristic (ROC) curves (AUC), specificity, sensitivity, accuracy, positive predictive value, and negative predictive values of the radiomics signature, clinical risk factors, and radiomics nomogram were calculated. Decision curve analysis (DCA) was performed to determine the clinical usefulness of the radiomics nomogram.

**Results:**

The LASSO analysis finally included 11 radiomics features, which were defined as the radiomics signature. The individualized prediction nomogram was constructed by integrating the radiomics signature, maternal age, and body mass index (BMI). The nomogram exhibited a good performance both in the training cohort [AUC of 0.89 (95% CI, 0.82–0.95)] and test cohort [AUC of 0.85 (95% CI, 0.73–0.97)] for predicting PE from GH. The DCA indicated that clinicians and patients could benefit from the use of radiomics nomogram.

**Conclusion:**

The radiomics nomogram could individually predict PE from GH. The nomogram could be conveniently used to facilitate the treatment decision for clinicians and patients.

## Introduction

As one of the important obstetric diseases, pre-eclampsia (PE) is the main cause of death of maternal and prenatal morbidity (Magee et al., 2022). The clinical manifestations of PE, namely, headache, vertigo, nausea, stomach pain, and vomiting. The common complications of PE include fetal growth restriction, iatrogenic premature birth, and chronic intrauterine hypoxia (Khan et al., 2006). Previous studies have shown that patients with PE had a higher risk of the heart, cerebrovascular disease, diabetes, and cognitive impairment in the long term (Aukes et al., 2012). At present, the only effective way to treat PE is to terminate the pregnancy. Therefore, the therapeutic significance is mainly to prevent the occurrence of PE.

At present, the diagnosis of PE is mainly based on gestational hypertension [GH, a rise of blood pressure (BP) ≥ 140/90 mm Hg at or after 20 weeks of gestation] accompanied by the presence of proteinuria (24-h urinary protein ≥ 300 mg) or the clinical manifestations (Brown et al., 2018). The 24-h urinary protein remains the gold standard for the diagnosis of PE (Rani et al., 2014). However, BP is in dispute as a criterion for the diagnosis of PE, because BP is affected by blood volume, which usually increases during pregnancy (Mustafa et al., 2012). Furthermore, the detection process of 24-h urinary protein is relatively time-consuming and laborious. The PE progresses rapidly. It is easy to lose the opportunity to extend the gestational week or bring severe complications to both mother and fetus. Sometimes, patients with severe PE may only have mild manifestations in the early stage (Ray and Park, 2018). PE is easy to be ignored in clinical practice. However, when patients with PE have neurological complications such as cerebral edema, and cerebral hemorrhage, the mortality can be as high as 70% (Logue et al., 2016).

To evaluate the neurological injury conditions, the most classic understanding is to perform an imaging examination in patients with PE (Bell, 2010). Magnetic resonance imaging (MRI) could be used as a non-radiation and non-invasive method for evaluation of the brain lesions. Previous MRI studies on PE mostly focused on posterior reversible encephalopathy syndrome (PRES) (Fugate et al., 2010). However, PRES has a wide disease spectrum, namely, hypertension, sepsis, nephropathy, and the patients using immunosuppressive agents (Dong et al., 2017). With the development of neuroimaging, researchers gradually found that the imaging manifestations of PE included not only PRES, but also cerebral edema, cerebral infarction, cerebral hemorrhage, and cerebral venous thrombosis (Brewer et al., 2013; Di et al., 2018). Therefore, PRES is limited in the diagnosis of PE.

Diffusion-weighted imaging (DWI) and the corresponding apparent diffusion coefficient (ADC) maps can be used as additional diagnostic tools in stroke and the brain tumors. A previous study has shown that the basal ganglia were frequently (60%) involved in patients with PE, with slightly hyperintense on DWI (Mueller-Mang et al., 2009). A recent study showed that lower cerebral blood volume and blood flow were measured in patients with PE in the basal ganglia by using the DWI intravoxel incoherent motion (IVIM) approach (Nelander et al., 2018). Radiomics can be used to extract many features from tomographic images *via* high-throughput computing and convert them into comprehensive quantified data (Gillies et al., 2016). DWI-based radiomics was considered a biomarker (Li et al., 2021). Combined analyses of the radiomics features and the clinical risk factors can produce a radiomics nomogram, which is becoming the most promising approach for individualized management (Li et al., 2021).

We assumed that the ADC-based radiomics nomogram could be used to distinguish PE from GH. However, no study exists about radiomics nomogram that combines radiomics features with clinical risk factors to predict PE from GH. Therefore, this study aimed to establish a radiomics nomogram based on brain DWI and ADC maps to predict PE from GH.

## Materials and methods

### Patients

The prospective study was approved by our Institutional Review Board (No: 2019-S34-01). Informed consents were obtained in all the cases. From October 2017 to September 2021, 156 patients with hypertensive disorders of pregnancy were continuously enrolled in the study prospectively. The diagnostic criteria for GH were as follows: (Magee et al., 2022) Systolic blood pressure ≥ 140 mm Hg and/or diastolic blood pressure ≥ 90 mm Hg first detected at/after 20 weeks of gestation; (Khan et al., 2006) Negative urine protein test. The diagnostic criteria for PE were as follows: patient diagnosed with GH with any of the following: (Magee et al., 2022) Urine protein quantification ≥ 300 mg/24 h; (Khan et al., 2006) Urine protein/creatinine ≥ 0.3; (Aukes et al., 2012) Positive random urine protein test; (Brown et al., 2018) Negative urine protein test, but with involvement of any organ such as heart, lung, liver, and kidney, or with an abnormal change of any system of the digestive system, nervous system, and blood system, or with involvement of the placenta/fetus.

**FIGURE 1:**
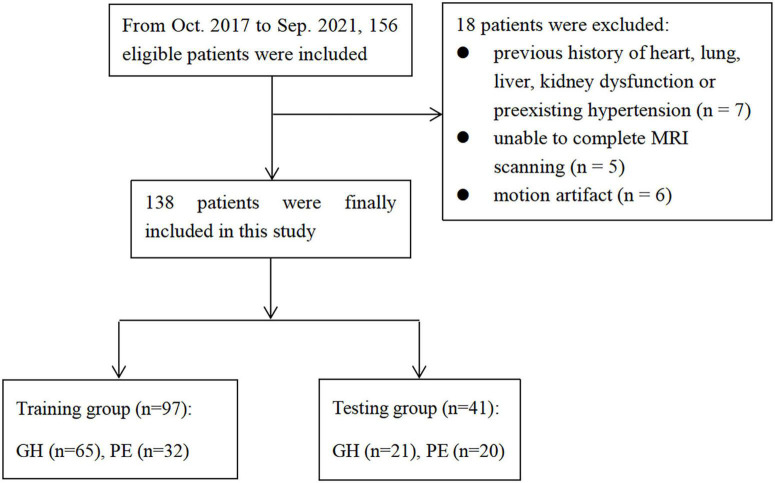
The workflow of this study.

The exclusion criteria were as follows: (Magee et al., 2022) Patients with a previous history of heart, lung, liver, kidney dysfunction, or pre-existing hypertension (*n* = 7); (Khan et al., 2006) Patients unable to complete MRI scanning (*n* = 5); (Aukes et al., 2012) Poor MRI image quality due to motion artifact (*n* = 6). At last, 58 patients with PE and 80 patients with GH after delivery were continuously included in the present study. The MRI scanning was performed within seven days after delivery. The patients were divided into a training cohort (*n* = 97) and a test cohort (*n* = 41) in a ratio of 7:3. The work flow is shown in Figure 1.

### Clinical characteristics

The alanine aminotransferase (ALT), aspartate aminotransferase (AST), total bilirubin (TB), and prothrombin time (PT) were examined in all the subjects. Urine volume and urine protein (UP) were recorded for 24 h. The patients’ maternal age, body mass index (BMI), diastolic pressure (DP), and systolic pressure (SP) were also recorded.

### Neuroimaging studies

All MRI were performed by a 3.0 *T* scanner (Verio, Siemens, and Erlangen, Germany). The patients lay in a supine position and breathed freely during the acquisition. The following sequences were obtained: axial vibe T1-weighted imaging (T1WI) with TR/TE = 3.4/1.3, thickness = 3 mm; turbo spin-echo (SE) T2-weighted imaging (T2WI) with TR/TE = 2,770/64, thickness = 4 mm. Axial echo planar imaging DWI with TR/TE = 7,100/79, thickness = 4 mm, and *b* values of 0 and 1,000 s/mm^2^. The ADC maps were automatically generated.

### Magnetic resonance imaging segmentation and radiomics features extraction

The radiomics data processing was performed referring to previous work (Yan et al., 2020). In brief, brain MRI from each patient was imported into ITK-SNAP.^1^ The DWI (*b* = 0 and *b* = 1,000) and ADC map were subsequently aligned to T2WI and T1WI. The region of interest (ROI, namely, putamen, and globus pallidus) was manually drawn on each slice of the DWI and ADC map referring to T2WI and T1WI by a radiologist (Reader 1, with 3 years of experience in neuroimaging) blinded to the patients’ clinical information. Overall, 1 week later, 30 patients were randomly chosen and the same manual drawings were repeated by Reader 1 and by another radiologist (Reader 2, with 12 years of experience in neuroimaging). The inter-observer and intra-observer correlation coefficients (ICCs) were calculated.

The MR imaging registration and extraction of radiomics features from the DWI and ADC maps were performed by using python (Version 3.9.2^2^) with the ‘‘Nipype’’ package and ‘‘pyradiomics’’ package, respectively. The images were normalized by subtracting from mean values and then dividing by SD. Then, a voxel array shift of 300 was used to ensure all gray level values were within a 0-600 range. At last, a fixed bin width of 1 was used to extract the features. The radiomics features extraction followed the IBSI recommendation. The differences between the pyradiomics and the IBSI recommendations were documented.^3^

### Radiomics signature and clinical risk factors selection in the training cohort

Radiomics features with either inter-observer or intra-observer ICC < 0.75 were considered unstable features. The features with a high correlation to another feature (Pearson’s correlation coefficients > 0.9) were considered redundant features (the one with the largest mean absolute correlation). Unstable and redundant features were dropped for further analysis. A binary least absolute shrinkage and selection operator (LASSO) logistic regression analysis with 10-fold cross-validation was used to select the radiomics features. The selected radiomics features were defined as the radiomics signature. The radiomics score (rad-score) for each patient was calculated by using a linear combination of the radiomics signature.

Multivariate binary logistic regression analysis was performed to select the clinical characteristics (clinical risk factors) for predicting PE in the training cohort.

### Radiomics nomogram building, testing, discrimination, and calibration

A radiomics nomogram for predicting PE from GH was developed by combining the radiomics signature with the clinical risk factors using multivariable logistic regression. The radiomics nomogram was validated in the testing cohorts. The area under the curves (AUCs) of receiver operator characteristic (ROC) was used to evaluate the discrimination performance of the radiomics nomogram in the training and test cohorts. The calibration curve was used to assess the goodness of fit of the radiomics nomogram in the training cohort by the bootstrap test method. A co-occurrence network was used to analyze the correlation between the radiomics signature and the clinical characteristics in the training cohort.

By quantifying the net benefits at different threshold probabilities in the training and test cohorts, decision curve analysis (DCA) was performed to determine the clinical usefulness of the radiomics nomogram.

### Statistical analysis

Statistical analysis was performed by R software (version 4.0.5^4^). Student *t*-test or Mann–Whitney *U*-test was used to compare the differences in continuous variables after the Shapiro–Wilk testing for normality. Chi-squared test was used to compare the differences in categorical variables. The association between the radiomics signature and clinical characteristics was assessed by Spearman’s correlation. The “glmnet” package was used for LASSO and logistic regression; the “icc” package was used for ICC calculation; the “caret” package was used for removing redundant features; the “rms” package was used for nomogram calculation; the “pROC” package was used for AUC; the “dca.R” package was used for DCA. The ROC curve analysis was performed to calculate the AUC and corresponding 95% CI. *P* value of less than 0.05 was considered statistically significant.

## Results

### Clinical characteristics

**FIGURE 2:**
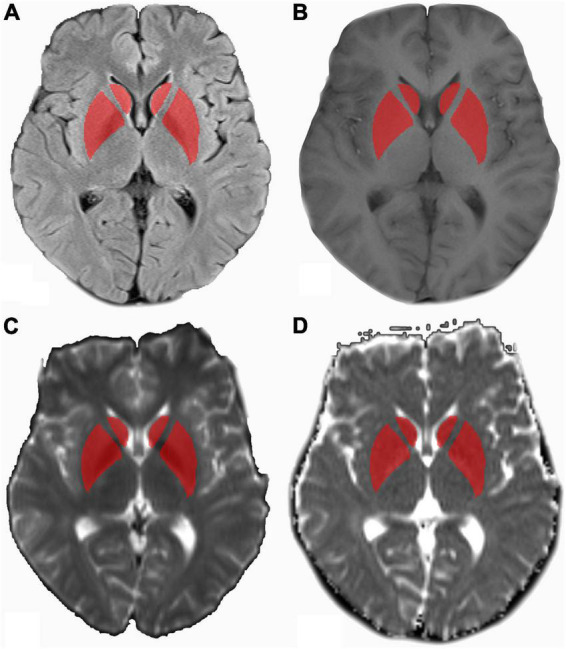
MR images in a 25-year-old patient with pre-eclampsia (PE). **(A)** Axial T2WI marked with ROI drawn on bilateral basal ganglia referring to axial T1WI **(B)**, axial diffusion-weighted imaging (DWI) (*b* = 1,000 s/mm^2^) **(C)**, and apparent diffusion coefficient (ADC) maps **(D)**. ADC, apparent diffusion coefficient; DWI, diffusion-weighted imaging; T1WI, T1-weighted imaging; T2WI, T2-weighted imaging; ROI, region of inter.

Bilateral symmetrical hyperintensity was found on T1-weighted imaging in globus pallidus in eight patients with PE. The hyperintensity was not seen after 1-month follow-up. The PRES was found in six patients with PE. The T2 hyperintensity disappeared 1 month later. White matter lesions were found in five patients with PE and in eight patients with GH. The maternal age, BMI, DP, and UP were different between patients with PE and GH. No significant difference in SP and liver function tests were shown. The clinical characteristics of the training and test cohorts are shown in Table 1. The ROI drawing is demonstrated in Figure 2.

**TABLE 1:** Clinical characteristics of the training and test cohort.

	Training cohort			Test cohort		
	GH (*N* = 65)	PE (*N* = 32)	*P*-value	GH (*N* = 21)	PE (*N* = 20)	*P*-value
Radscore	0.23 (0.1)	0.52 (0.2)	< 0.001	0.33 (0.2)	0.65 (0.22)	< 0.001
Age	27 (4.0)	30 (4.8)	0.003	26 (3.4)	29 (5.3)	0.035
BMI	27.1 (3.0)	31.3 (4.7)	< 0.001	27.1 (3.6)	30.1 (3.7)	0.013
SP	139 (7.3)	143 (12.4)	0.077	141 (8.9)	140 (11.0)	0.845
DP	85 (8.5)	91 (10.8)	0.006	87 (11.7)	92 (6.9)	0.059
UP	0.08 (0.1)	0.62 (0.9)	0.003	0.08 (0.1)	0.49 (0.9)	0.046
ALT	13.6 (6.5)	13.1 (7.3)	0.749	12.8 (5.3)	14.4 (7.7)	0.452
AST	17.7 (7.0)	18.0 (6.3)	0.801	18.8 (9.8)	18.3 (5.9)	0.833
PT	10.4 (1.3)	10.4 (0.6)	0.738	10.0 (0.7)	10.1 (0.5)	0.585
TB	8.3 (3.6)	7.98 (3.9)	0.671	8.5 (4.5)	8.0 (3.1)	0.699
ALB	35.2 (2.7)	34.0 (2.9)	0.067	33.9 (3.3)	34.3 (2.4)	0.667

ALB, albumin; ALT, alanine aminotransferase; AST, aspartate aminotransferase; BMI, body mass index; DP, diastolic pressure; GH, gestational hypertension; PE, Pre-eclampsia; PT, prothrombin time; TB, total bilirubin; SP, systolic pressure; UP, 24 h urinary protein.

### Feature selection and radiomics signature construction

**FIGURE 3:**
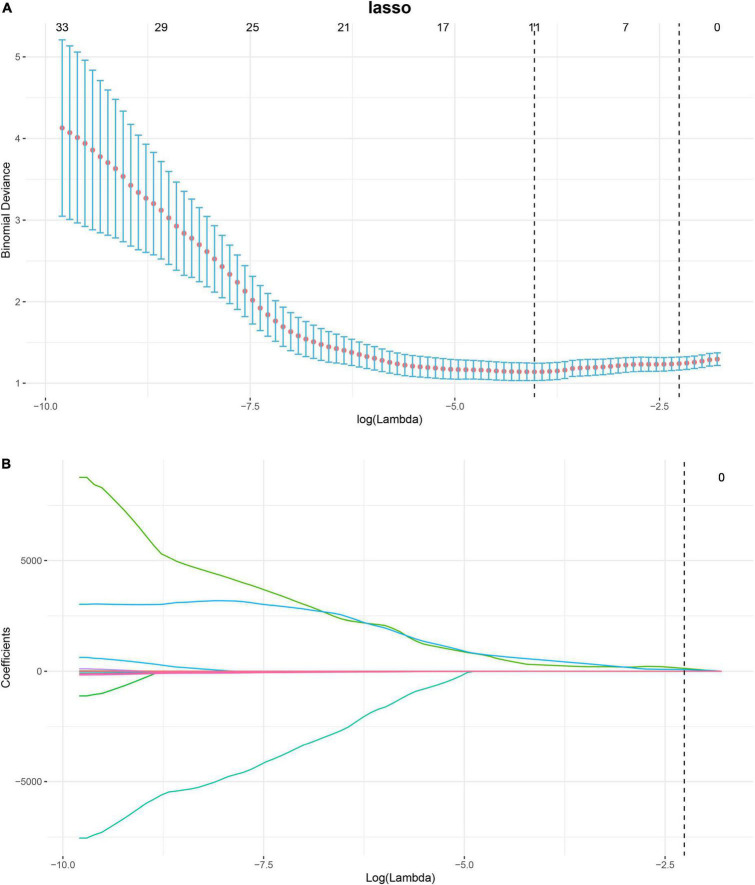
Process of feature selection in patients with pre-eclampsia (PE) by binary least absolute shrinkage and selection operator (LASSO) logistic regression. In total, eleven non-zero coefficients (radiomics signature) with the highest curves (AUC) for predicting PE are selected **(A)**. The coefficients of each radiomics feature are plotted *vs*. log (lambda) **(B)**. AUC, area under curve; LASSO, least absolute shrinkage and selection operator.

Based on the images feature extracting, 293 radiomics features were extracted, namely, 14 shape features, 54 first-order features, and 225 texture features. After removing features with either inter-observer or intra-observer ICC < 0.75, Pearson correlation coefficients > 0.9, 103, and 34 features were retained, respectively. The LASSO analysis finally included 11 radiomics features, which was defined as the radiomics signature. The mean cross-validated error and the estimated standard error were 1.24 ± 0.079 (Figure 3). The rad-score calculation is as follows: Rad-score = 0.30968 + −1.75917 × shape Elongation + 0.03582 × shape LeastAxisLength + −0.00411 × shape MinorAxisLength + 0 × ADC glcm ClusterProminence + 61.27831 × ADC glcm JointEnergy + −0.20626 × ADC glszm Large Area Low Gray Level Emphasis + 0.05289 × ADC glszm ZoneVariance + 102.62058 × ADC ngtdm Coarseness + 0.00108 × b0 first-order Median + 0.00113 × b1000 first-order Range + 3e-05 × b1000 gldm Small Dependence High Gray Level Emphasis.

### Radiomics nomogram development and validation

**FIGURE 4:**
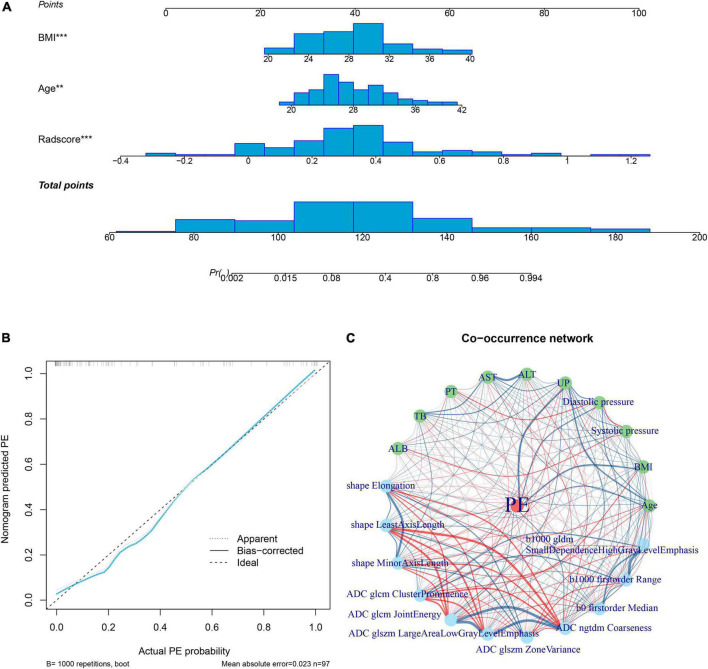
Radiomics nomogram. The radiomics nomogram is developed by integrating rad-score with maternal age and body mass index in the training cohort; the distributions are shown on the nomogram lines **(A)**. The calibration curve shows the goodness of fit of the nomogram by the bootstrap test method (mean absolute error = 0.023) **(B)**. A co-occurrence network shows the correlations between radiomics signature and clinical risk factors of PE **(C)**. ALB, albumin; ALT, alanine aminotransferase; AST, aspartate aminotransferase; BMI, body mass index; PE, Pre-eclampsia; PT, prothrombin time; TB, total bilirubin; UP, 24 h urinary protein.

Multivariate logistic regression analysis showed that the maternal age and BMI were clinical risk factors for PE (Supplementary Table 1). Therefore, a radiomics nomogram was constructed by integrating the radiomics signature, maternal age, and BMI (Figure 4). The calibration curves showed good discrimination performances of the radiomics nomogram (Figure 4). A co-occurrence network showed the correlation between clinical characteristics and radiomics signatures in patients with PE (Figure 4).

### The performance of the rad-score, clinical risk factors, and radiomics nomogram

The AUCs of rad-score were 0.83 (95% CI: 0.75–0.92) and 0.84 (95% CI: 0.72–0.96) in the training cohort and test cohort, respectively. The AUCs of clinical risk factors were 0.80 (95% CI: 0.69–0.90) and 0.83 (95% CI: 0.70–0.96) in the training cohort and test cohort, respectively. The AUCs of radiomics nomogram were 0.93 (95% CI: 0.88–0.98) and 0.89 (95% CI: 0.78–0.99) in the training and test cohort, respectively. The AUC, specificity, sensitivity, positive predictive value, and negative predictive values are shown in Table 2.

**TABLE 2:** Diagnostic performance of the training and test cohorts.

		AUC	95% CI	SPE	SEN	NPV	PPV	*P* *	*P*+
Training cohort	Radiomics	0.83	0.75–0.92	0.77	0.78	0.88	0.63	–	0.611
	Clinical risk factors	0.80	0.69–0.90	0.95	0.62	0.84	0.87	0.611	–
	Nomogram	0.93	0.88–0.98	0.88	0.88	0.93	0.78	0.002	0.022
Test cohort	Radiomics	0.84	0.72–0.96	1.00	0.60	0.72	1.00	–	0.898
	Clinical risk factors	0.83	0.70–0.96	0.86	0.75	0.78	0.83	0.898	–
	Nomogram	0.89	0.78–0.99	0.95	0.70	0.77	0.93	0.143	0.289

AUC, area under curve; NPV, negative predictive value; PPV, positive predictive value; SEN, sensitivity; SPE, specificity.

*vs. radiomics by DeLong test; +vs. clinical risk factor by DeLong test.

### Clinical usefulness

**FIGURE 5:**
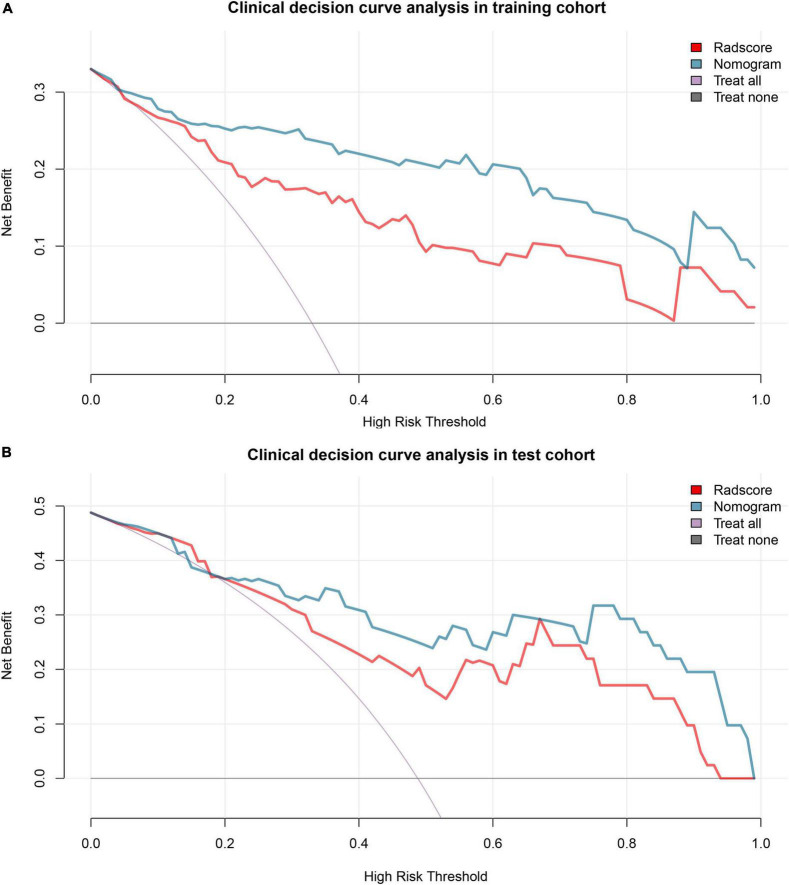
Decision curve analysis. Decision curve analysis shows that the radiomics nomogram adds net benefit in predicting pre-eclampsia (PE) both in the training cohort **(A)** and test cohort **(B)** than treat all the patients as PE (purple line) or as gestational hypertension (GH) (black line). GH, gestational hypertension; PE, pre-eclampsia.

The decision curve analysis showed that both the radiomics signature and radiomics nomogram could add a net benefit to the patients. When the threshold probability was within a range from 0 to 100%, the net benefit of using the nomogram to predict PE was more than the treat-all or treat-none scheme in both the training and test cohort (Figure 5).

## Discussion

Our preliminary study showed that the radiomics signature based on the DWI and ADC maps had high-diagnosis performance in predicting PE from GH. It indicated that this computer-based data analysis could be used as a helpful diagnosis tool to detect the presence of PE in patients with GH.

The worldwide incidence of hypertension in pregnancy is about 10%, the etiology, and pathogenesis of which have always been the focus of attention and research in related fields of obstetrics and gynecology (American College of Obstetricians and Gynecologists; Task Force on Hypertension in Pregnancy, 2013). At present, the diagnosis of PE is mainly based on increased blood pressure and large amounts of proteinuria. Clinical risk factors for PE were summarized in a previous study (Chappell et al., 2021). Pregestational diabetes, maternal age, multifetal pregnancy, and BMI > 30 kg/m^2^ were the main clinical risk factors of new-onset PE without chronic medical conditions. High BMI and high-maternal age were clinical risk factors for women at risk of PE, who were recommended for aspirin prophylaxis (Chappell et al., 2021). A previous study showed that PE was more frequent in women with obesity compared with those with normal weight (Pfaller et al., 2021). Another study including a large sample of singleton pregnancies reported that advanced maternal age (≥ 40 years) was associated with an increased risk of PE but not GH (Guarga et al., 2021). In this study, maternal age and BMI were identified as the clinical risk factors for predicting PE from GH, which is in accordance with the previous studies.

The pathogenesis of PE is not clear. No good diagnostic criteria are available for PE (Roberts and Redman, 1993). Clinically, PE is characterized by neurological symptoms, such as convulsions, epilepsy, visual disturbance, and consciousness disturbance. MRI is a safe and non-invasive examination, which is useful in detecting brain lesions (Sengar et al., 1997). The most typical MRI manifestation was white matter lesions in posterior circulation in patients with PE (Sanders et al., 1991; Schaefer et al., 1997). However, white matter lesions could also be found in women with normal pregnancies (Wiegman et al., 2014). In recent years, neuroimaging studies found that gray matter would be affected at the same time (Casey et al., 2000). Furthermore, the imaging findings were not limited to the brain area of the posterior circulation, but could also involve the frontal lobe and basal ganglia (Lee et al., 2008). A previous study showed that visual brain lesions on T2WI were significantly higher in patients with PE/eclampsia, and the basal ganglia region was more frequently (60%) involved (Mueller-Mang et al., 2009).

The DWI with ADC is a technique of calculating a diffusivity value to quantitatively assess the water molecule movement. In tissues, the Brownian motion of water molecules causes phase dispersion, resulting in attenuation of the measured signal intensity on the DWI. A previous study showed that abnormalities in DWI were found in patients with PE with cytotoxic edema (Shah and Whitty, 1999). Nelander et al. used IVIM to assess the whole brain perfusion and edema in patients with PE. The IVIM technique is based on the separation of true diffusion from perfusion-related pseudodiffusion using a DWI sequence with multiple *b*-values. The results showed that basal ganglia were the only area affected in patients with PE and slightly hyperintense on DWI was shown (Nelander et al., 2018). Therefore, in this study, we placed the area of interest in the bilateral basal ganglia.

The radiomics converts the medical images into high-dimensional data by high-throughput extraction of quantitative features (Li et al., 2021). Nomogram is a diagnostic model that combines imaging and clinical information to determine an individualized prediction or treatment decision, and prognosis evaluation. In this study, a radiomics nomogram was developed by integrating the radiomics signature with clinical risk factors of patients with PE. The radiomics nomogram was confirmed to have the ability to generate a personalized probability to predict PE in patients with GH. The shape features reflected a volume change in the basal ganglia in patients with PE. The ADC- and DWI-related radiomics features reflected the change of ADC values and DWI signal in the basal ganglia in patients with PE. The changes mentioned earlier might be led by higher cerebral perfusion and brain edema. The microvasculature of the basal ganglia with a higher number of non-anastomotic vessels is also part of the explanation for these changes (Euser and Cipolla, 2007). In the past, multi-predictive models have been developed to screen first trimester PE (with delivery at < 34 weeks’ gestation) and preterm PE (with delivery at < 37 weeks’ gestation). Most of the previous studies made risk assessments in first-trimester screening (De Kat et al., 2019). Better accuracy in the prediction of first trimester PE was achieved compared with preterm PE (Chaemsaithong et al., 2022). However, first trimester PE affects only 0.5% of all the pregnancies (Lisonkova and Joseph, 2013). And the prediction models of first trimester PE usually have a low-positive predictive value (Lisonkova and Joseph, 2013). Furthermore, most studies chose the strategies to distinguish PE in normal pregnant women. In this study, a radiomics nomogram was used to predict preterm PE from GH. By combing the radiomics features and clinical risk factors, the radiomics nomogram generated better diagnostic performance to distinguish PE from GH than using the clinical risk factors alone.

In addition, we applied a decision curve analysis, which offers insight into clinical consequences on the basis of threshold probability, and the net benefit of the radiomics nomogram in predicting PE was calculated. The net benefit is defined as the proportion of true positives minus the proportion of false positives, weighted by the relative harm of false-positive and false-negative results. Our results indicated the good clinical usefulness of radiomics nomogram.

There were some limitations of this study. First, all of the subjects underwent MRI after delivery. A previous study showed that the DWI hyperintensity with decreased ADC in patients with PE indicated irreversible brain lesions, which persisted for 8 weeks after delivery (Loureiro et al., 2003). Another study showed that the brain lesions of PE would continue after pregnancy for 5 and 15 years (Siepmann et al., 2017). Second, this was a single-center study with a limited sample size. Therefore, our results should be validated from multiple centers with a larger sample size prior to clinical implementation. Third, most patients did not receive follow-up MRI examinations. Subsequent changes in the basal ganglia lesions could not be evaluated.

In conclusion, the radiomics nomogram could individually predict PE from GH. The nomogram could be conveniently used to facilitate the treatment decision for clinicians and patients.

## Data availability statement

The raw data supporting the conclusions of this article will be made available by the authors, without undue reservation.

## Ethics statement

The studies involving human participants were reviewed and approved by Institutional Review Board of Jinshan Hospital, Fudan University. The patients/participants provided their written informed consent to participate in this study.

## Author contributions

M-DL and YL: designed the research study. M-DL, J-JL, and A-RZ: performed the research. X-FL and YL: analyzed the data and wrote the manuscript. YL and J-WQ: supervisors of this study. All authors read and approved the final manuscript.

## Footnotes

1
http://www.itksnap.org

2
https://www.python.org/

3
https://pyradiomics.readthedocs.io/

4
http://www.Rproject.org
