# Supplementary material for: Prediction of pre-eclampsia by using radiomics nomogram from gestational hypertension patients
Source: Front Neurosci. 2022 Aug 5;16:961348. doi: 10.3389/fnins.2022.961348 (PMC9389207; doi:10.3389/fnins.2022.961348)
Supplement: Supplementary file 1 [file Table_1.docx]

Supplementary Table 1. Multivariate binary logistic regression analysis in selecting the clinical characteristics

|  | Estimate | Std. | Error | t | P |
| --- | --- | --- | --- | --- | --- |
| (Intercept) | -3.1349 | 1.2780 | -2.4530 | 0.0162 | * |
| Age | 0.0267 | 0.0097 | 2.7490 | 0.0073 | ** |
| BMI | 0.0457 | 0.0102 | 4.4650 | 0.0000 | *** |
| SP | 0.0082 | 0.0050 | 1.6340 | 0.1059 |  |
| DP | 0.0086 | 0.0045 | 1.9110 | 0.0593 |  |
| ALT | 0.0033 | 0.0077 | 0.4340 | 0.6651 |  |
| AST | -0.0067 | 0.0074 | -0.9080 | 0.3666 |  |
| PT | -0.0252 | 0.0386 | -0.6540 | 0.5149 |  |
| TB | -0.0142 | 0.0125 | -1.1400 | 0.2575 |  |
| ALB | -0.0007 | 0.0168 | -0.0390 | 0.9692 |  |

ALB, albumin; ALT, alanine aminotransferase; AST, aspartate aminotransferase; BMI, body mass index: DP, diastolic pressure; PT, prothrombin time; TB, total bilirubin; SP, systolic pressure.

***, < 0.001; **, < 0.01, * < 0.05. AIC = 106.75.
